# All Three Supersystems—Nervous, Vascular, and Immune—Contribute to the Cortical Infarcts After Subarachnoid Hemorrhage

**DOI:** 10.1007/s12975-024-01242-z

**Published:** 2024-04-30

**Authors:** Jens P. Dreier, Alexander Joerk, Hiroki Uchikawa, Viktor Horst, Coline L. Lemale, Helena Radbruch, Devin W. McBride, Peter Vajkoczy, Ulf C. Schneider, Ran Xu

**Affiliations:** 1 Center for Stroke Research Berlin, Campus Charité Mitte, Charité–Universitätsmedizin Berlin, corporate member of Freie Universität Berlin, Humboldt-Universität zu Berlin, and Berlin Institute of Health, Charitéplatz 1, 10117 Berlin, Germany; 2 Department of Experimental Neurology, Charité–Universitätsmedizin Berlin, corporate member of Freie Universität Berlin, Humboldt-Universität zu Berlin, and Berlin Institute of Health, Berlin, Germany; 3 Department of Neurology, Charité–Universitätsmedizin Berlin, corporate member of Freie Universität Berlin, Humboldt-Universität zu Berlin, and Berlin Institute of Health, Berlin, Germany; 4 Bernstein Center for Computational Neuroscience Berlin, Berlin, Germany; 5 Einstein Center for Neurosciences Berlin, Berlin, Germany; 6 Department of Neurology, Jena University Hospital, Jena, Germany; 7 Barrow Aneurysm & AVM Research Center, Barrow Neurological Institute, St. Joseph’s Hospital and Medical Center, Phoenix, AZ USA; 8 Institute of Neuropathology, Charité – Universitätsmedizin Berlin, corporate member of Freie Universität Berlin, Humboldt-Universität zu Berlin, and Berlin Institute of Health, Berlin, Germany; 9 The Vivian L. Smith Department of Neurosurgery, McGovern Medical School, The University of Texas Health Science Center at Houston, Houston, TX USA; 10 Department of Neurosurgery, Charité – Universitätsmedizin Berlin, corporate member of Freie Universität Berlin, Humboldt-Universität zu Berlin, and Berlin Institute of Health, Berlin, Germany; 11 Department of Neurosurgery, Cantonal Hospital of Lucerne and University of Lucerne, Lucerne, Switzerland; 12 DZHK, German Centre for Cardiovascular Research, Berlin, Germany

**Keywords:** Stroke, Subarachnoid hemorrhage, Delayed cerebral ischemia, Neuromonitoring, Spreading depolarization, Vasospasm

## Abstract

The recently published DISCHARGE-1 trial supports the observations of earlier autopsy and neuroimaging studies that almost 70% of all focal brain damage after aneurysmal subarachnoid hemorrhage are anemic infarcts of the cortex, often also affecting the white matter immediately below. The infarcts are not limited by the usual vascular territories. About two-fifths of the ischemic damage occurs within ~ 48 h; the remaining three-fifths are delayed (within ~ 3 weeks). Using neuromonitoring technology in combination with longitudinal neuroimaging, the entire sequence of both early and delayed cortical infarct development after subarachnoid hemorrhage has recently been recorded in patients. Characteristically, cortical infarcts are caused by acute severe vasospastic events, so-called spreading ischemia, triggered by spontaneously occurring spreading depolarization. In locations where a spreading depolarization passes through, cerebral blood flow can drastically drop within a few seconds and remain suppressed for minutes or even hours, often followed by high-amplitude, sustained hyperemia. In spreading depolarization, neurons lead the event, and the other cells of the neurovascular unit (endothelium, vascular smooth muscle, pericytes, astrocytes, microglia, oligodendrocytes) follow. However, dysregulation in cells of all three supersystems—nervous, vascular, and immune—is very likely involved in the dysfunction of the neurovascular unit underlying spreading ischemia. It is assumed that subarachnoid blood, which lies directly on the cortex and enters the parenchyma via glymphatic channels, triggers these dysregulations. This review discusses the neuroglial, neurovascular, and neuroimmunological dysregulations in the context of spreading depolarization and spreading ischemia as critical elements in the pathogenesis of cortical infarcts after subarachnoid hemorrhage.

## Introduction

Aneurysmal subarachnoid hemorrhage (aSAH) affects about 30,000 people annually in the USA alone and has a high rate of morbidity and mortality [1]. In the recent prospective, observational, multicenter, diagnostic phase III trial DISCHARGE-1, in aSAH patients, cumulative focal brain damage from intracerebral hemorrhage (ICH), early cerebral ischemia (ECI), and delayed cerebral ischemia (DCI) up to day 14, as determined by neuroimaging, was the best predictor of patient outcome half a year after the initial hemorrhage [2]. Ninety-five of 180 patients (52.8%) had ICH. The mean cumulative volume of damage due to ICH was 19 ± 29 ml across all patients. Damage due to ECI occurred in 123 of 180 patients (68.3%); the mean volume of damage was 27 ± 67 ml. The early focal brain injury (EBI), composed of ICH and ECI, amounted to 46 ± 73 ml and affected 151 of 180 patients (83.9%). Ten patients died early. Delayed infarcts occurred in 98 of the 170 early survivors (57.6%), and their mean volume was 36 ± 80 ml across all early survivors. The relatively high rate of delayed infarcts in DISCHARGE-1 might have two reasons: (1) the better image quality of MRI compared to CT, typically used in standard clinical studies, and (2) the fact that the neurosurgeons virtually always included patients with large amounts of subarachnoid blood [2].

Importantly, DCI is a potentially modifiable cause of brain damage during neurocritical care because treatment can begin prior to the onset of damage. Since almost 50% of focal brain damage in DISCHARGE-1 was due to DCI, the patient outcome could have been improved if there had been both an effective therapeutic strategy for DCI and, equally important, an effective strategy to automatically detect DCI in order to target therapeutic interventions. Automated real-time detection of DCI is particularly important as most high-risk patients are comatose, and therefore, neurological deterioration cannot be detected clinically. In DISCHARGE-1, for example, 90/170 early survivors (52.9%) were not clinically assessable during the entire observation period of 2 weeks. To make matters worse, these comatose patients also had a significantly greater delayed infarct volume than the clinically assessable patients [2].

In order to develop effective diagnostic and therapeutic strategies, it is important to better understand the pathophysiology of focal brain damage and in particular that of cortical infarcts after aSAH. This review is therefore intended to provide an overview of this complex pathophysiology, taking into account all three supersystems—nervous, vascular, and immune.

## The Large Majority of Early and Delayed Infarcts After aSAH Are Cortical

The primary objective of DISCHARGE-1 was to calculate (i) sensitivity and (ii) specificity for a known cut-off value for the peak total spreading depolarization (SD)-induced depression duration of a recording day (PTDDD) during the delayed neuromonitoring period (PTDDD_delayed_) that indicates delayed ischemic infarcts ipsilateral to the recording strip and (iii) to estimate a new cut-off value (https://doi.org/10.1186/ISRCTN05667702) [2]. The SDs were measured with a subdural electrode strip placed via craniotomy or burr hole trepanation.

In Horst et al. [3], the early and delayed infarct volumes in a subpopulation of 136 DISCHARGE-1 patients on the side of the subdural electrode strip were examined in more detail. The volume of early infarcts involving the cortex was significantly larger than the volume of early deep infarcts (7.3 ± 22.3 ml versus 1.2 ± 3.2 ml, *p* = 0.002, Mann–Whitney rank sum test (MWRST)). The difference between cortical and deep infarcts was even greater in delayed infarcts (17.8 ± 44.5 ml versus 1.3 ± 5.4 ml, *p* < 0.001, MWRST). In other words, 86% of the early infarcts and 93% of the delayed infarcts involved the cortex. Early and delayed cortical infarcts correlated significantly with each other (Spearman 0.19, *p* = 0.032, *n* = 136), while early and delayed deep infarcts did not correlate with each other (Spearman 0.02, *p* = 0.811, *n* = 136). Pathophysiological processes leading to early cortical infarcts could therefore also be relevant for delayed cortical infarcts, whereas early and delayed deep infarcts are etiologically rather different. Overall, it is estimated that 69% of the total bilateral focal brain damage after aSAH was due to early or late ischemic infarcts involving the cortex, 23% to ICH, and 8% to deep ischemic infarcts. Two-fifths of the ischemic infarcts occurred early, and three-fifths were delayed.

Cortical ischemic lesions also represented the predominant pathomorphological pattern of parenchymal damage in previous neuroimaging and autopsy studies in aSAH patients [4–8]. Regarding the etiology of these infarcts, it is interesting to note that Stoltenburg-Didinger and Schwarz [8] found intravascular thrombi in only four of 106 patients with cortical infarcts in their autopsy study, which never preceded the infarcts but were typical of secondary microcirculatory disturbances within the necrotic area. Endothelial swelling could be excluded as an etiology because this occurs only temporarily and would, if at all, obstruct the lumina of capillaries. Also, compression was ruled out as a potential cause since the subarachnoid blood clot would lead to venous compression prior to compression of arteries. The cortical infarcts, however, were never primarily hemorrhagic, but they were typically anemic. In the autopsy studies, no relationship was found between the cortical ischemic lesions and angiographic vasospasm in either humans or non-human primates [6, 9].

**Fig. 1:**
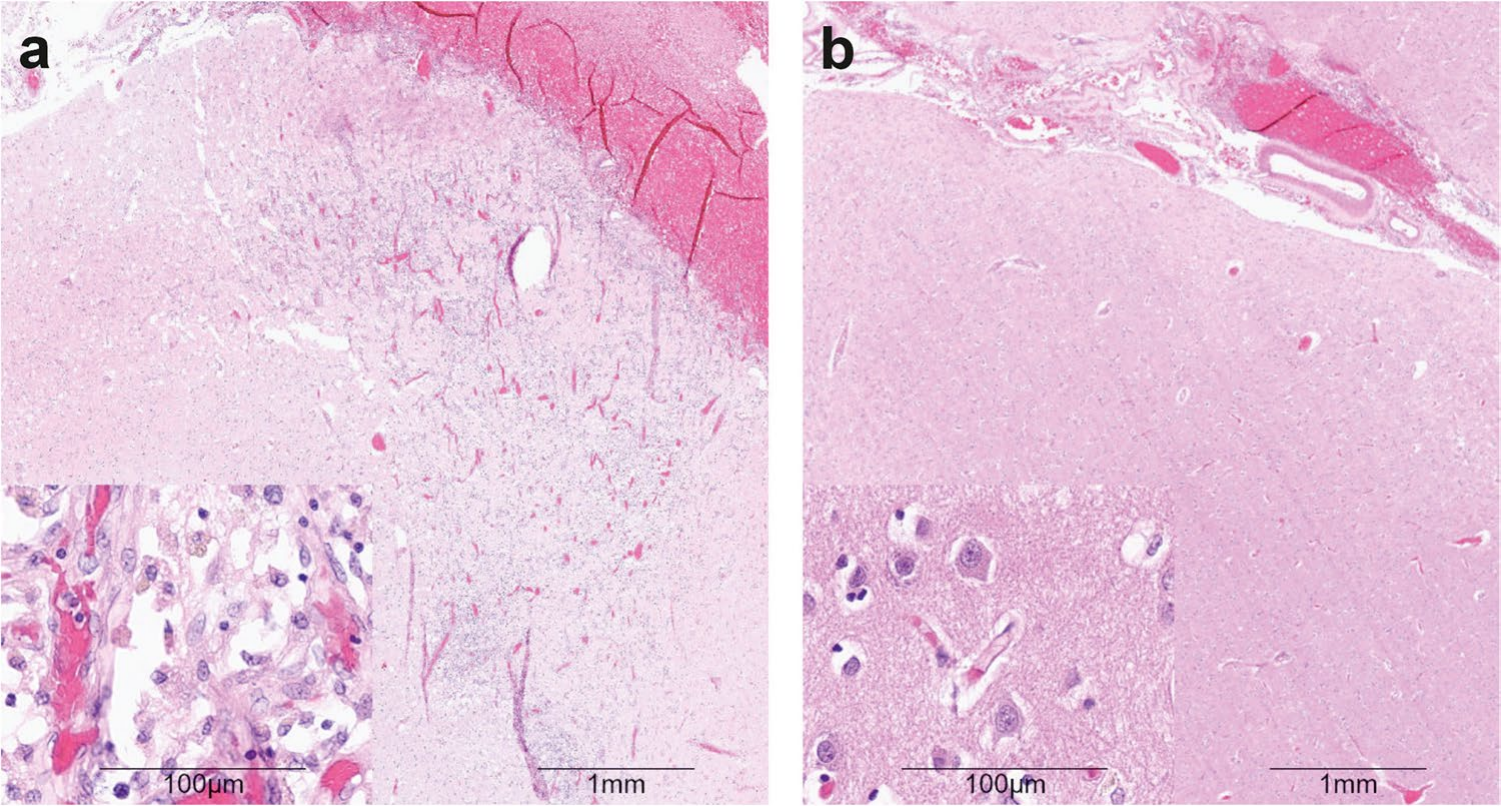
Autopsy case of an 80-year-old female patient with subarachnoid hemorrhage resulting from the rupture of an anterior communicating artery aneurysm. On admission, the patient was comatose and showed signs of decerebrate rigidity (World Federation of Neurosurgical Societies (WFNS) [212] scale 5). The initial CT demonstrated basal subarachnoid hemorrhage with involvement of the ventricles (modified Fisher grade 4 [213]). She remained comatose during the further clinical course and died 25 days after the initial hemorrhage under palliative care. The autopsy revealed an extensive subarachnoid hemorrhage with a punctum maximum in the basal cisterns. The cerebral convexities were also partially covered with blood. Specimens were taken at predefined locations, formalin-fixed and paraffin-embedded. **a**, **b** Hematoxylin and eosin-stained sections of the left frontolateral cortex. This area was covered with subarachnoid blood. **a** A wedge-shaped cortical irregularity with its base at the cortical surface. At higher magnification (inset), massive infiltration of macrophages, extensive neo-vascularization, and neuronal loss were seen. These findings are consistent with a subacute cortical infarct adjacent to a thick sulcal blood clot (right upper corner) (scale bar = 1 mm; scale bar inset = 100 µm). **b** Normal appearing cortex adjacent to a thinner sulcal blood clot (right upper corner). At higher magnification (inset), the normal neuronal somata are clearly visible (scale bar = 1 mm; scale bar inset = 100 µm)

**Fig. 2:**
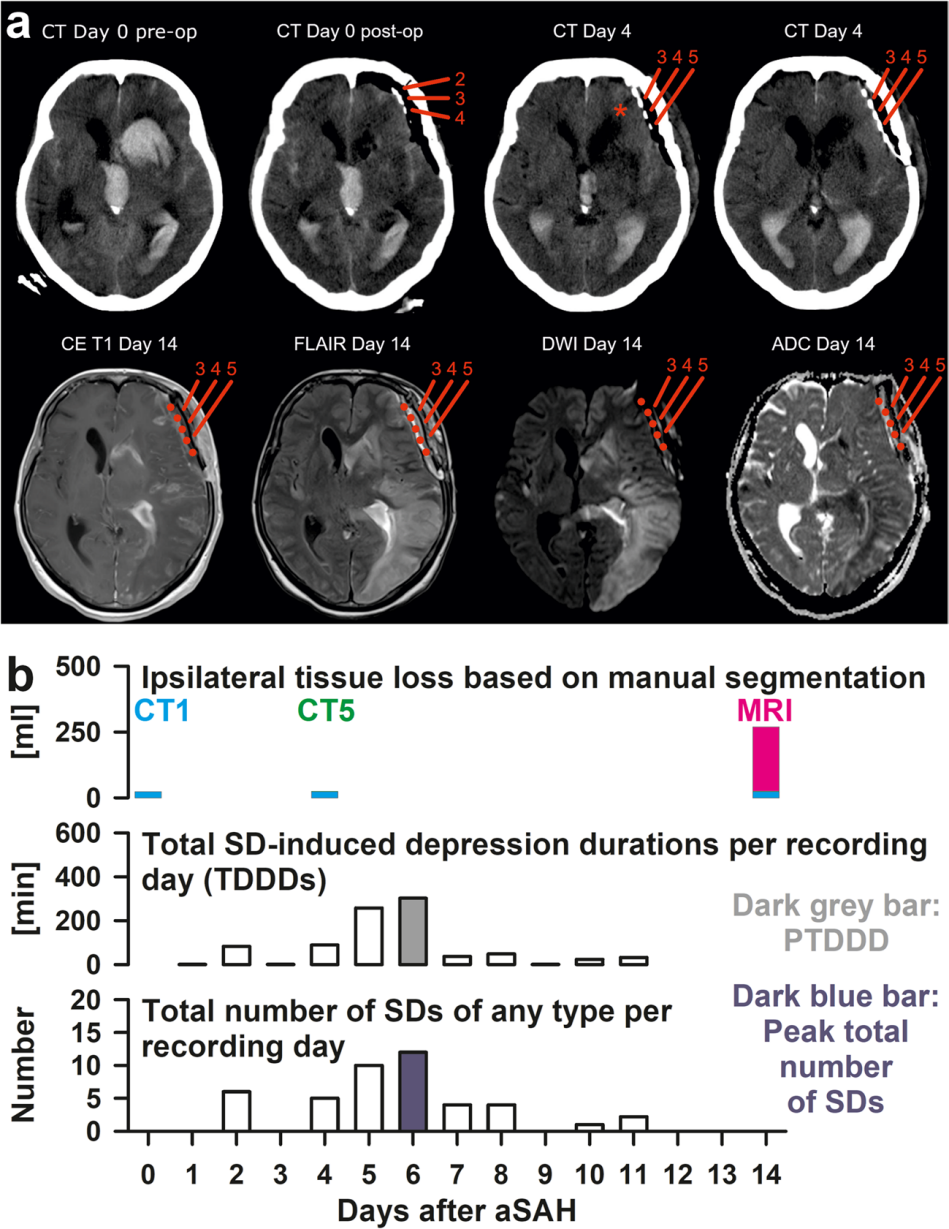
**a** Representative CT and MR images of a 61-year-old female patient with subarachnoid hemorrhage resulting from rupture of an aneurysm of the left middle cerebral artery (MCA). All images were aligned to the T1 scan of the MRI on day 14 after the initial hemorrhage. The first three CT images from the left show an axial section at the level of the third ventricle. The CT image in the top row on the far right and the MR images in the bottom row show a section of the brain at the level of the basal ganglia (+ 6 mm in the direction of the vertex in comparison to the first three CT images). The initial CT on admission (CT day 0 pre-op = CT1 in **b**) demonstrated subarachnoid hemorrhage and massive intraventricular hemorrhage that extended into the left cerebral parenchyma. On the same day, a second CT was performed after surgical clipping of the aneurysm, placement of a subdural electrode strip over the left frontolateral cortex, and evacuation of the intracerebral hematoma. Electrodes 2–5 are marked in red. Electrodes 1 and 6 are not shown. The CT (CT day 0 post-op = CT2) revealed perifocal edema surrounding the evacuated intracerebral hemorrhage. Furthermore, the left frontal cortex showed a subtle hypodensity between electrodes 2 and 3. The hypodensity only became clearly visible on the CT of day 4 (= CT5 in **b**) (asterisk). These findings are consistent with a cortical infarct developing in the early phase after the initial hemorrhage. Of note, CT imaging on day 4 (far right image in the upper panel) already demonstrated some sulcal effacement in the left hemisphere. On day 7, digital subtraction angiography (DSA) was performed (not shown). Visual assessment of the left arteriogram yielded mild vasospasm of the internal carotid artery and posterior circulation (basilar artery, P1 and P2 segment of the left posterior cerebral artery (PCA)). Moderate vasospasm was found in the proximal and distal segments of the MCA and the anterior cerebral artery. MR imaging on day 14 showed contrast enhancement of the early infarcted cortex between electrodes 2 and 3 (CE T1 day 14). Posteriorly, the cortex of the left insula and the left operculum adjacent to electrodes 4–6 showed marked increase in signal intensity compared to the right hemisphere on fluid-attenuated inversion recovery imaging (FLAIR day 14) and a decrease in signal intensity on T1 imaging (CE T1 day 14). The interpretation of these signal alterations is challenging. As pseudonormalization occurs on images of the apparent diffusion coefficient (ADC) in cerebral infarction after ~ 10 days [214], the lack of ADC alterations in the left insular and opercular cortex may indicate subacute infarction that developed around day 4. However, this is somewhat contradicted by the fact that no contrast enhancement of the cortex was seen (CE T1 day 14), which is typical of infarction after ~ 10 days [214]. We therefore favor the diagnosis of incomplete infarction in the left insular and opercular cortex adjacent to electrodes 4–6. Further posteriorly, FLAIR imaging (FLAIR day 14) and diffusion-weighted imaging (DWI day 14) showed a large hyperintense area that was hypointense in the ADC images and included the left posterior MCA territory and the part of the convexity supplied by the left PCA, including the occipital pole [215]. In other words, this delayed infarct involving gray and white matter was not limited by the boundaries of the normal vascular territories. It may be added that the DSA showed no fetal-type PCA. Ancillary findings were small scattered delayed cerebral infarcts in the right MCA territory and a mixture of cytotoxic and vasogenic edema surrounding the evacuated intracerebral hemorrhage. **b** Time course of focal brain damage and spreading depolarization (SD)-variables in the same patient as in **a**. The upper row 1 in **b** shows the progression of focal brain damage from CT1 to CT5 to the MRI on day 14 based on manual neuroimage segmentation of the hemisphere ipsilateral to the recording strip [2]. Rows 2 and 3 below show the time course of the SD variables: For each day, SDs were counted, and depression durations were scored to determine the total duration of SD-induced activity depression per recording day (TDDD) (row 2) and the total number of SDs per recording day (row 3). The peak TDDD (PTDDD) and peak SDs/day (peakSD) were defined for each patient as the maximal values among all recording days (indicated as a dark gray and dark blue bar, respectively). As can be seen, the delayed SD cluster began on day 4 and reached its maximum on day 6, i.e., in the temporal phase in which the delayed infarct development can be assumed on the basis of the neuroimaging in **a**

A consistent autoptic finding was that the cortical lesions typically occurred below subarachnoid blood clots, suggesting that local blood products were involved in their pathogenesis [8, 9] (Fig. 1). This corresponds with the clinical observation that thick layers of subarachnoid blood on admission CT scans are consistently among the most important predictors of infarction and unfavorable outcome after aSAH [3, 10–15]. The autopsy study by Stoltenburg-Didinger and Schwarz [8] was published in 1987 which was before the International Cooperative Study on the Timing of Aneurysm Surgery [16]. As such, most patients did not receive any treatment of the aneurysm (156/207 patients, 75.4%) which is much higher than is typical in recent studies. Interestingly, the ratio of autopsy cases with cortical to territorial infarcts was 13:1 in patients who had not undergone clip ligation, whereas it was 3:1 in patients who had undergone surgery due to a relative increase in the incidence of territorial infarcts (*p* < 0.001, chi-square test). In the pathoanatomical descriptions, the cortical infarcts are typically bell-shaped, corresponding to the territory of small perforating arteries, or laminar, corresponding to the territories of rectangular branches of the cortical arteries [4, 6, 8, 9]. Overall, pathologists suggest that the most likely etiology of these infarcts is spasm and not (micro)thrombosis [8], with the relevant spasms affecting the cortical rather than the proximal arteries [6, 8]. However, in DISCHARGE-1, many large delayed infarcts were not only purely laminar but also affected the underlying white matter (Fig. 2a). In this context, it is important to note that spasms of the arteries in the cerebral cortex can lead not only to infarcts in the cortex but also to infarcts in the underlying white matter, since the arteries that supply the white matter first pass through the cortex before reaching the white matter [17–19]. Vasoconstriction at the level of the cortex can therefore interrupt the blood supply not only to the cortex but also to the underlying white matter. Another important feature of these infarcts is that they are not limited by the usual territorial boundaries (Fig. 2a).

In accordance with these pathomorphological details, severe repetitive vasospastic events lasting up to 2.5 h were recorded in the cerebral cortex of patients with aSAH [20, 21]. To demonstrate this, optoelectrodes placed directly over newly developing infarcts were used. The newly developing infarcts were proven with longitudinal neuroimaging. These acute vasospastic events were triggered by SDs, which are briefly explained below.

## SD

**Fig. 3:**
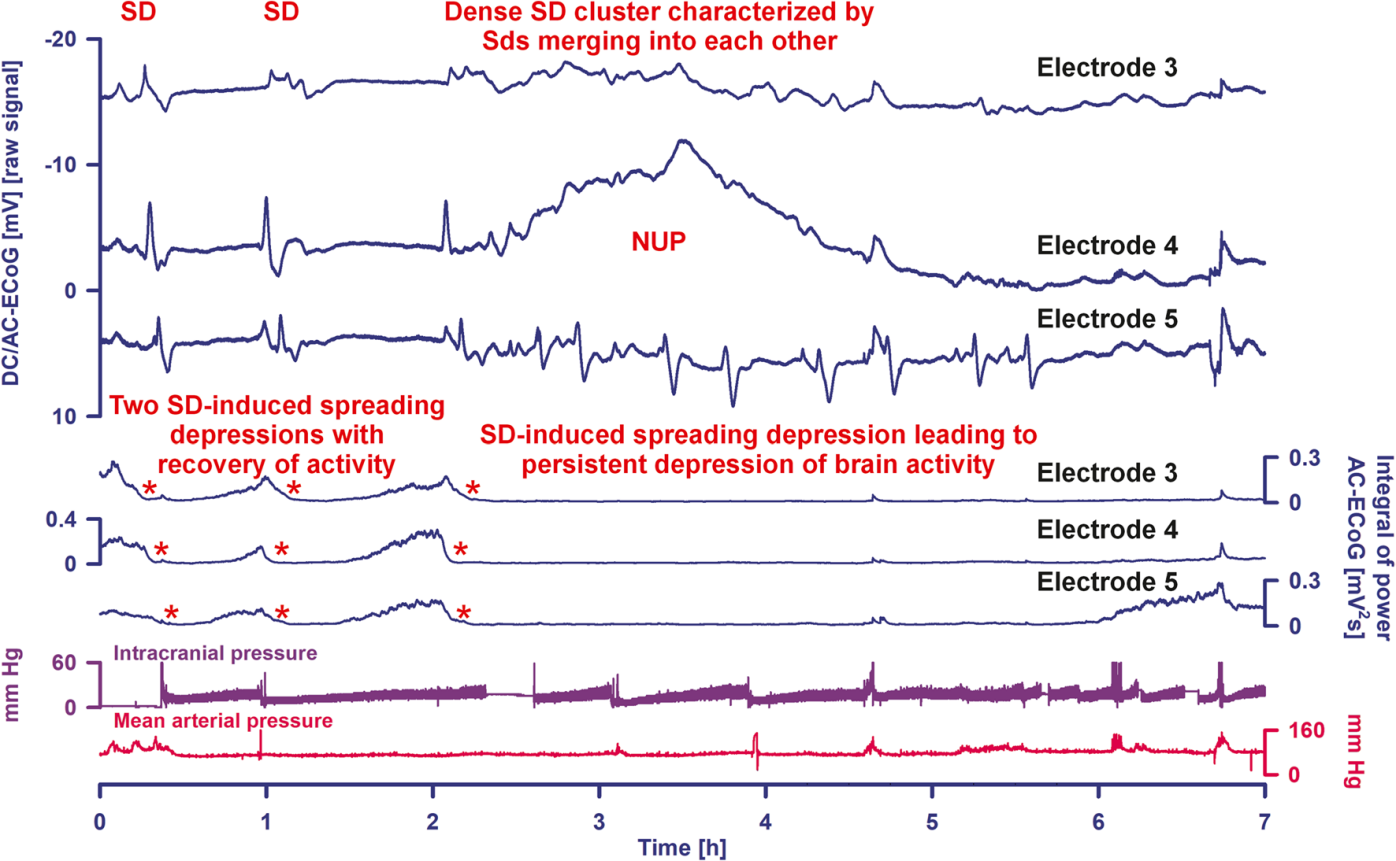
A cluster of spreading depolarizations (SD) precedes the development of the large delayed ischemic infarct that is shown in Fig. 2 between the CT scan on day 4 and the MRI scan on day 14. Traces 1–3 give the raw direct current (DC)/alternating current (AC)-electrocorticography (ECoG) recordings (band-pass, 0–45 Hz), demonstrating the propagation of the negative DC shifts along the cortex from electrode to electrode, which identify the SDs. The ECoG traces are oriented according to the convention of electroencephalography (EEG) with negativity up and positivity down. The distance between two neighboring electrodes is always 1 cm. This section of the cluster begins at the start of day 6 with three separate SDs, the first of which starts at electrode 3, the second at electrode 5, and the third at electrode 4. The third SD leads to a compaction of the cluster with SDs that merge into one another at electrodes 3 and 4. At electrode 4 and to a certain extent also at electrode 3, these later SDs are superimposed on a negative ultraslow potential (NUP) (current sink), while the SDs at electrode 5 remain more clearly separated from each other, retain their high amplitudes, and are superimposed on a positive ultraslow potential (current source). The traces at electrodes 3 and 4 are therefore typical for an area where ischemic damage develops, while the trace at electrode 5 is typical for a more peripheral area of damage development [20]. This is also supported by the changes in spontaneous brain activity. The depressive effect of the SDs on the spontaneous activity is assessed in traces 4–6 using the integral of the power in the AC frequency band between 0.5 and 45 Hz (red asterisks mark the onsets of SD-induced spreading depression) [99]. While there is a recovery of spontaneous brain activity after the first two SDs at all three electrodes, the third SD leads to a persistent depression of spontaneous activity. In contrast to electrodes 3 and 4, there is then a spontaneous recovery of brain activity at electrode 5 a little less than 4 h after the start of the third SD, which supports the hypothesis that the electrophysiological events at this electrode were less severe than at electrodes 3 and 4. The whole course of the SD-induced depressions is shown in Fig. 2b. The intracranial pressure (ICP) was measured via an external ventricular drain (EVD) (trace 7) and the arterial pressure via a catheter in the radial artery (trace 8). The fluctuations in the ICP result from short-term opening of the EVD

SD is characterized by near-complete breakdown of the transmembrane ion gradients, cytotoxic edema, and sustained near-zero depolarization of neurons [22–25]. Neurons lead the SD with astrocytes following [26, 27]. SD is typically observed as a large negative direct current (DC) shift [28] (Fig. 3). In alternating current (AC)-electrocorticography (ECoG), SD classically triggers a rapidly developing reduction in the amplitudes of spontaneous activity, known as spreading depression [29, 30] (Figs. 2b and 3).

To prevent the neuronal network from dying in the SD state, it must repolarize. In order for it to repolarize, sufficient ATP has to be available to activate the membrane pumps and in particular the Na,K-ATPases [31–34]. However, repolarization must occur before the so-called commitment point of SD is reached, at which point irreversible damage to neurons begins [35]. For severe focal cerebral ischemia, the commitment point is reached after about 20 min [20]. For complete circulatory arrest, it is reached earlier [35–40]. SDs that lead to mass neuronal damage typically show the transition to a negative ultraslow potential (NUP) [20, 41] (Fig. 3).

## Primary Focal Ischemia Triggers SD, While SD Can Trigger Secondary Focal Ischemia

**Fig. 4:**
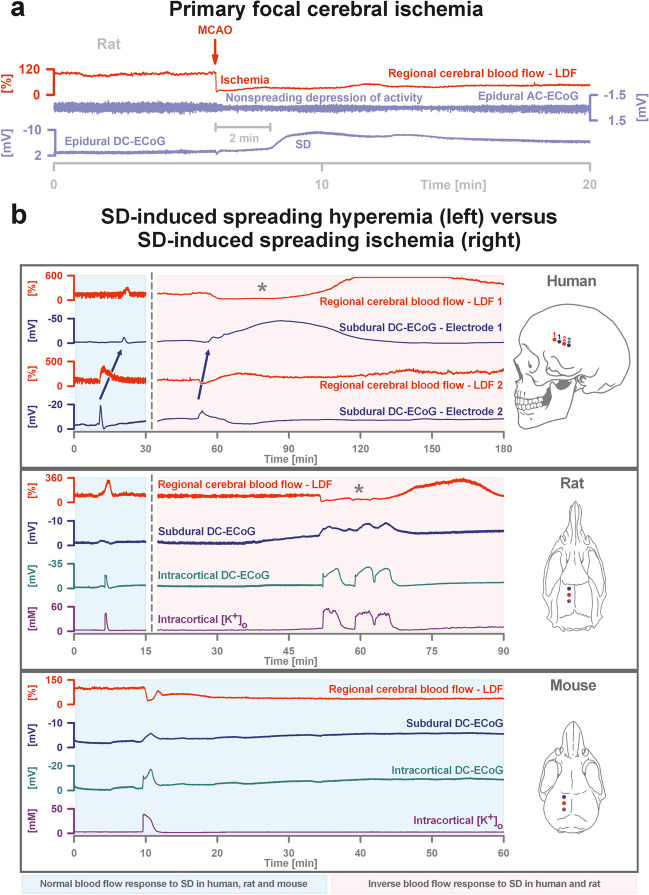
**a** The characteristic pathophysiological sequence of events in the rat after filament occlusion of the middle cerebral artery (MCAO). Trace 1 from top to bottom gives regional cerebral blood flow (rCBF). The first reaction to filament occlusion is the steep drop in rCBF. Trace 2 shows the spontaneous brain activity using alternating current (AC)-electrocorticography (ECoG) (band-pass, 0.5–45 Hz). The primary focal ischemia triggers a rapidly developing reduction in the amplitudes of spontaneous brain activity within a few seconds, which typically begins practically simultaneously in the entire ischemic region (= nonspreading depression of activity) [2, 20, 23]. The ECoG traces are oriented according to the convention of electroencephalography (EEG) with negativity up and positivity down. Trace 3 gives an epidural direct current (DC)/AC-ECoG recording (band-pass, 0–45 Hz) where spreading depolarization (SD) is observed as a large negative DC shift with a delay of 1 min after the onset of the primary focal ischemia. These original recordings emphasize again that in primary focal ischemia, the first SD in the region of minimal perfusion typically occurs 1 min or later after the onset of ischemia, as there is obviously still sufficient ATP for the membrane pumps to prevent SD in the first minute(s) [42, 216–220]. In addition, trace 2 shows that SD can no longer trigger spreading depression of spontaneous activity in the region of minimal perfusion, since spontaneous activity is already depressed by the previous occurrence of nonspreading depression of activity. **b** Normal rCBF responses to SD in naïve human, rat, and mouse (B57BL/6) cortex (left panels, light blue) and inverse rCBF responses to SD in human and rat cortex with disturbed neurovascular unit (NVU) (right panels, pink). In naïve human cortex, SD (dark blue arrow between negative DC shifts) induces predominant hyperemia (laser-Doppler flowmetry (LDF)) and lasts only a short time. In contrast, the panel at the top right shows an SD inducing a characteristic drop in rCBF typical of spreading ischemia (asterisk) after aneurysmal subarachnoid hemorrhage (aSAH). The spreading ischemia lasted for 50 min followed by marked, long-lasting hyperemia. Note that the durations of the negative DC shifts correlate well with the durations of the SD-induced hypoperfusions at the two different recording sites because decrease in perfusion and energy supply limits Na,K-ATPase activity and prolongs the depolarization [23]. The spreading ischemia was recorded on day 9 after aSAH [20]. The patient developed a delayed infarct at the recording site between two CT scans on days 8 and 12. On day 13, she died from the progressive brain infarctions. The panels below show that the phenomenologies of both normal spreading hyperemia and spreading ischemia in rats are indistinguishable from those in humans. In the rat, spreading ischemia resulted from an aSAH-mimicking model based on NO deprivation and elevated baseline extracellular potassium concentration [23, 72, 106]. The rCBF response to SD in naïve mouse cortex appears to start from a high baseline level and occupies an intermediate position between the normal and inverse responses of phylogenetically higher mammals. Extracellular potassium was recorded here with an ion-sensitive microelectrode

Figure 4a shows the standard sequence of a sudden drop in regional cerebral blood flow (rCBF) followed by a nonspreading depression of spontaneous neuronal activity several seconds and SD about a minute after middle cerebral artery occlusion (MCAO) in a rat (filament occlusion). The figure thus shows an example of a typical primary ischemia that triggers SD in the ischemic, still fully viable ischemic center after a latency period of 1–5 min or even longer [42, 43]. The first SD then slowly migrates outwards from the site of origin [44]. This standard sequence of primary ischemia, which triggers SD many minutes before irreversible neuronal damage has occurred, and gradually spreads from the ischemic center to the periphery, is nicely illustrated, for example, in video 1 by Zhao et al. [43] using imaging of intracellular calcium after photothrombosis. Importantly, the first ischemia-induced SD cannot initiate spreading depression in the ischemic center and inner penumbra because these zones have already been subject to nonspreading depression and activity cannot be further depressed (Fig. 4a). In animals, the standard sequence of primary ischemia is basically the same in cardiocirculatory arrest: a drop in rCBF followed by a nonspreading depression of brain activity a short time later and SD with a latency of minutes. This sequence of events has also been observed in patients with cardiocirculatory arrest during neurocritical care [36].

Although ischemia is an important trigger for SD, it is by no means the only one. Rather, there is a plethora of different triggers that are more or less pathological [33]. One of the standard triggers used experimentally, for example, is an increased extracellular potassium concentration [45]. Yet, electrographic seizures can also act as a trigger for SD [46–49], and there are even genetic conditions that increase the likelihood of SD and in some cases have been proven to lead to spontaneous SDs [50–55]. Of particular interest in the context of aSAH is that decreased nitric oxide availability (NO) occurs after aSAH via different mechanisms [23, 56–62] and that decreased NO availability lowers the threshold for SD in both animals and brain slices [63, 64]. It has been hypothesized that NO acts in this way because it modulates calcium entry through P/Q-type calcium channels and N-methyl-d-aspartate receptors (NMDAR) in cortical neurons [65]. In vivo, the reduction in rCBF due to NO depletion should additionally favor the occurrence of SD.

In otherwise normal tissue of phylogenetically higher mammals such as rats, swine, and humans, SD acts as a strong stimulus to increase rCBF (spreading hyperemia = normal hemodynamic response) (left panels in Fig. 4b). After repolarization, this increase is typically followed by physiological, long-lasting, moderate hypoperfusion (= oligemia) [21, 66–70]. Under these conditions, SD is short-lived and harmless [71]. However, if the neurovascular coupling is disturbed, SD can cause severe vasoconstriction instead of vasodilation (= inverse hemodynamic response) [23, 72]. This means that a long-lasting local perfusion deficit occurs during the depolarization phase (= spreading ischemia), which prevents repolarization of the tissue and can ultimately lead to a brain infarct (Dreier et al., 1998; Dreier et al., 2000) (right panels in Fig. 4b). A fact relevant to many genetic models is that the rCBF response to SD in mice under physiological conditions is already slightly shifted towards an inverse response [54, 73, 74] (Fig. 4b).

SD-induced spreading ischemia is due to SD-induced spasm of pial and cortical arteries, arterioles, and most likely also proximal capillary segments [23]. This acute vasospastic event is currently the most severe form of vasospasm known. It occurs spontaneously, spreads in the cerebral cortex, affects the entire microcirculation, extends proximally at least to the pial arteries, and can occur several times in succession, whereby the duration can be prolonged. Tissue in which the SD passes through experiences drastic and rapid (within seconds) rCBF drop. Since the vascular supply to the white matter is via the cortical circulation [17–19], the white matter under the cortex may also become ischemic [75]. SD-induced spreading ischemia in aSAH patients may last from several tens of seconds to at least 2.5 h and is often followed by high-amplitude, persistent hyperemia, which may then revert to oligemia [20, 21, 76] (Fig. 4b). In aSAH patients, SD-induced spreading ischemia leading to cerebral infarction in the area of the probes in longitudinal neuroimaging started at a median partial pressure of oxygen (p_ti_O_2_) of 12.5 mmHg (interquartile range (IQR), 9.2–15.2) in the brain tissue [20], which is already below the normal range [77]. As ischemia spread, p_ti_O_2_ then fell further to 3.3 mmHg (IQR, 2.4–7.4). Similarly, rCBF showed a downward trend even before the onset of SD-induced spreading ischemia. Immediately before the onset of spreading ischemia leading to infarction, rCBF was 57% (IQR, 53–65) compared to baseline and then dropped to 26% (IQR, 16–42) during spreading ischemia [20].

In summary, SD is the characteristic response of the assembly of neurons, astrocytes, and other cell types to primary focal ischemia in the cerebral cortex before they die, but SD can also start in non-ischemic or mildly ischemic cortical tissue and cause severe spreading ischemia as a form of secondary ischemia. SD-induced spreading ischemia rather than primary ischemia is typically observed during the development of delayed infarcts after aSAH [2, 20, 21, 23, 72] (Fig. 4b).

## PTDDD_delayed_

As a result of SD-induced spreading ischemia, the ATP level decreases even more than during normal SD [31, 32], the Na,K-ATPase activity necessary for tissue repolarization correspondingly lacks, and both the SD and the SD-associated depression of neuronal activity are prolonged. Accordingly, the duration of SD-induced spreading ischemia strongly correlates with the durations of both the SD and the activity depression [21, 78] (Fig. 4b). However, when comparing the two, the zone of persistent activity depression is always much larger than that of depolarization [79], which could be related, for example, to the increased release of adenosine in a large radius around the ischemic zone [80].

**Fig. 5:**
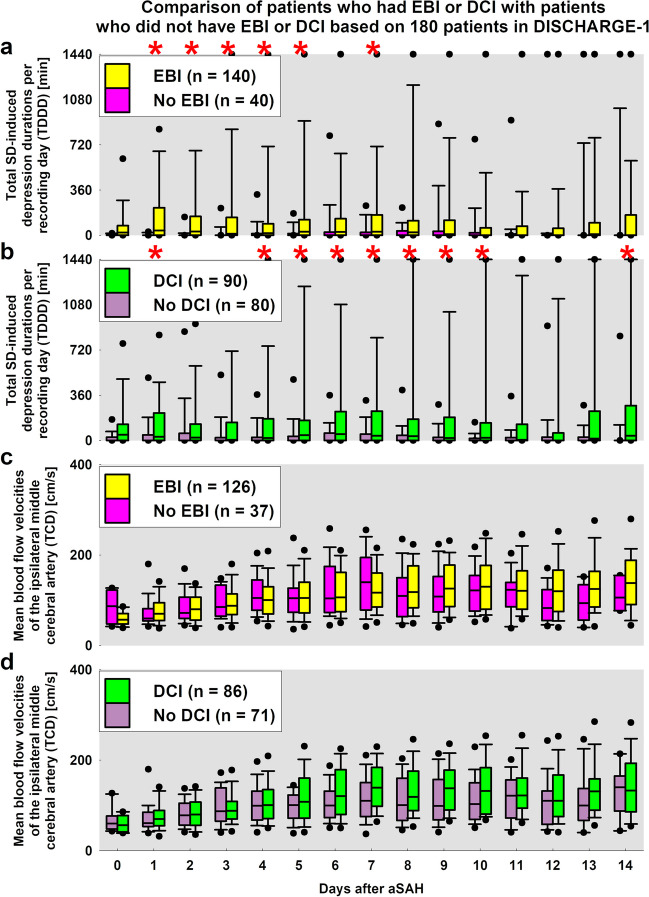
For each recording day, the respective total duration of activity depression induced by spreading depolarization (SD) of a recording day (TDDD) was compared between **a** patients with focal brain damage due to early brain injury (EBI) on the one hand and patients without focal brain damage due to EBI on the other hand and between **b** patients with focal brain damage due to delayed cerebral ischemia (DCI) on the one hand and patients without focal brain damage due to DCI on the other hand using Mann–Whitney rank sum tests and post-hoc Bonferroni correction. EBI was composed of focal brain damage due to intracerebral hemorrhage and early cerebral ischemia. Red asterisks indicate significant results after strict Bonferroni correction. **c** The comparison of mean blood flow velocities in the middle cerebral artery (MCA) ipsilateral to the subdural recording strip, measured by transcranial Doppler sonography (TCD), between patients with focal brain damage due to EBI compared to patients without focal brain damage due to EBI. **d** The comparison of mean blood flow velocities in the MCA between patients with focal brain damage due to DCI compared to patients without focal brain damage due to DCI. After strict Bonferroni correction, we could no longer detect any significant differences between the two groups compared in **c** and **d**, respectively. However, without strict Bonferroni correction, the mean velocities on the 15 days (= 15 tests) in **d** showed statistical differences between *p* = 0.0500 and *p* = 0.0033 on each day from day 6 to day 10 (= 5 tests). With 15 tests, only one uncorrected significant result is to be expected by chance. Since we observed significance in 5 out of 15 tests and then also on consecutive days, and considering that the statistical hypothesis tested is related to the same basic hypothesis—mean velocities correlate with delayed infarcts—one could argue that the Bonferroni correction is too conservative in this case. However, even without strict Bonferroni correction, it remains the case that the association of delayed infarcts with ECoG-measured TDDDs was stronger than their association with TCD-measured mean blood flow velocities

Accordingly, PTDDD_delayed_ was the strongest predictor of DCI (reversible delayed neurological deficit or delayed infarct) in DISCHARGE-1 [2]. Based on the analyses in Horst et al. [3], PTDDD_delayed_ correlated with delayed cortical infarcts (Spearman 0.54, *p* < 0.001, *n* = 136) but not with delayed deep infarcts (Spearman 0.10, *p* = 0.234, *n* = 136). Conversely, angiographic vasospasm correlated with delayed cortical infarcts (Spearman 0.27, *p* = 0.006, *n* = 106) and with delayed deep infarcts (Spearman 0.20, *p* = 0.044, *n* = 106). Delayed cortical and deep infarcts correlated with each other (Spearman 0.22, *p* = 0.009, *n* = 136). Figure 5 shows the time course of the total SD-induced depression durations per recording day (TDDD) in DISCHARGE-1 patients with EBI compared to patients without EBI (Fig. 5a) and in patients with delayed infarcts compared to patients without delayed infarcts (Fig. 5b). While TDDDs were significantly higher in patients with EBI compared to patients without EBI in the first half of the neuromonitoring period, patients with delayed infarcts had significantly higher TDDDs on days 1, 5–11, and 14 compared to patients without delayed infarcts. These results fit quite well with the respective time periods of EBI and DCI but also show some overlap. That is, EBI appears to possibly induce SDs and SD-induced depression during the first week after the initial hemorrhage. Such an aftereffect of EBI complicates the detection of new delayed infarcts. However, all SDs after aSAH may be a marker for potential damage development. For example, patients with at least one SD in DISCHARGE-1 had an overall 3.1-fold increased relative risk and a 42% increased absolute risk of a poor outcome half a year after the initial hemorrhage [2]. Thus, depending on the type of intervention, it may not be necessarily critical to know whether the damage to an existing lesion progresses [79, 81] or whether new lesions develop elsewhere in order to decide if a therapeutic intervention should be performed. This assessment receives additional indirect support from the fact that the mechanisms of early and delayed cortical infarct development also appear to overlap [20, 82].

Figure 5 shows the time course of a surrogate marker for proximal vasospasm, namely the mean blood flow velocities of the middle cerebral artery (MCA) ipsilateral to the subdural electrodes measured daily by transcranial Doppler sonography (TCD). The observation that there were no significant differences in mean velocities between patients with EBI and patients without EBI was to be expected (Fig. 5c). However, there was also no significant difference in mean velocities between patients with delayed infarcts and patients without delayed infarcts on any day (Fig. 5d).

In the DISCHARGE-1 population, angiographic vasospasm was found to be a statistical mediator between intraventricular blood volume and delayed infarct volume, but it showed a weaker overall correlation with delayed infarct volume than the delayed SD variables [3]. The delayed SD variables were a significant mediator between subarachnoid blood volume and delayed infarct volume [3]. Angiographic vasospasm and delayed SD variables did not correlate with each other [2]. Although it is likely that the chronic increase in vascular tone throughout the arterial tree down to the capillaries is relevant for rCBF decline before SD-induced spreading ischemia and should additionally favor a greater rCBF decline during spreading ischemia [3, 83], the lack of correlation between angiographic vasospasm and delayed SD variables fits well with the clinical studies in which angiographic vasospasm could be effectively controlled pharmacologically, but without achieving a resounding success in the prophylaxis of DCI [84–87]. In other words, although the absence of angiographic vasospasm is statistically favorable, its presence alone does not seem to be sufficient to explain DCI. This is also supported by the result of the only randomized trial of mechanical and pharmacological angioplasty, which recently showed no reduction in delayed infarcts and a significantly worse patient outcome [88]. Oral nimodipine remains the only drug that has been shown to reduce the risk of DCI although it has no detectable effect on angiographic vasospasm at the dosage used [89–91]. However, the effect of oral nimodipine is certainly not sufficient. Accordingly, practically all patients in DISCHARGE-1 were treated with oral nimodipine, but damage due to DCI occurred in 98 of 170 early survivors (57.6%) [2].

## Animal Models of ECI and DCI

A model of delayed neurological deficits and cortical infarcts after injection of blood into the subarachnoid space was first described in dogs over 60 years ago [92]. Non-human primates exposed to subarachnoid blood clots also showed virtually identical cortical infarcts below the clots as aSAH patients [9]. Specifically for ECI after aSAH, the swine sulcal clot model exists as a model in which subarachnoid blood clots alone are sufficient to induce SDs and adjacent cortical infarcts in the early period [82]. This model might be pathophysiologically more relevant for human ECI than endovascular puncture models in rodents, which, similar to MCAO, lead to primary territorial ischemia and on this basis to early SDs [93]. Typical rodent models of experimental SAH use cisternal injection of blood or vessel puncture and convincingly replicate the evolution of angiographic vasospasm, but fail to replicate the characteristic clinical course of delayed infarcts in patients [94]. Accordingly, the cisternal injection model of blood in mice did not result in SDs either [95], and endovascular puncture failed to show spontaneous SDs in the delayed phase [96, 97]. The limitation of rodent models for DCI is that thick subarachnoid blood clots on admission CT scans are consistently among the most important predictors of delayed infarcts [3, 10–15], but the small lissencephalic brains of rodents, unlike the larger gyrencephalic brains of non-human primates, swine, and dogs, do not permit thick subarachnoid clots required for infarction. The conclusion is that although experimental SAH in rodents is on the one hand important for the investigation of selected aspects, it is on the other hand limited because, unlike in patients, delayed SD clusters and delayed infarcts as shown in Figs. 1, 2, and 3 do not occur [94, 98].

## Possible Mechanisms of Inverse Hemodynamic Responses

Even if the mesoscopic level of delayed infarct development in the human brain has been largely clarified on the basis of neuromonitoring technology [20, 21, 99], the question remains as to why exactly SDs on the one hand and inverse rCBF responses on the other occur after human aSAH [23]. An interesting observation is that although NO depletion alone was not sufficient to cause full-blown SD-induced spreading ischemia in previous rodent studies [72, 100, 101], no experimental protocol has yet been found that resulted in SD-induced spreading ischemia but simultaneously did not cause NO depletion [34, 72, 102, 103]. In this context, it may be mechanistically relevant that the vasodilator NO is well suited to attenuate the vasoconstrictor effects of an increase in free cytosolic calcium, since in both neuronal and endothelial nitric oxide synthase (NOS), calmodulin binding is caused by an increase in free cytosolic calcium with a half-maximal activity between 200 and 400 nM. When calmodulin affinity to NOS increases, it facilitates electron flow from NADPH in the reductase domain to heme in the oxygenase domain, thereby increasing NO synthesis [104]. SD causes a strong increase in cytosolic calcium in various cell types [105]. Accordingly, SD was found to induce NO synthesis in neurons and endothelial cells [64]. Many studies have shown that NO depletion is a characteristic feature after SAH [23, 56–62].

In addition to NO depletion, at least one additional experimental condition is required to produce SD-induced spreading ischemia [23, 72]. This second condition may ultimately be a decrease in α_2_ activity of Na,K-ATPase leading to increased calcium uptake by internal stores of astrocytes, vascular myocytes, and pericytes due to a decrease in calcium efflux via the plasmalemmal sodium/calcium exchanger [34, 106]. Increased calcium mobilization from internal stores during SD should then increase vasoconstriction and thus contribute to spreading ischemia if, in addition, the antagonistic effect of NO is absent [34].

Another interesting point is that inverse (vasoconstrictive) rCBF responses can be triggered not only by SDs in aSAH patients [20, 21], in animal models mimicking conditions after aSAH [34, 63, 72, 78, 106–110] and after experimental SAH [97]. Rather, they can also be triggered by (1) electrographic seizures in aSAH patients [111], and, after experimental SAH, by (2) functional activation in vivo [112] and by (3) electrical field stimulation in brain slices [113]. These observations may be mechanistically relevant although the extent of vasoconstriction in response to seizures, functional activation, or electrical stimulation is far less pronounced than in SD-induced spreading ischemia, consistent with the fact that, for example, the increase in cytosolic calcium under these conditions is also much less than during SD [33, 105, 114].

Calcium is an important second messenger in many cells, including astrocytes. Astrocytic processes encase more than 90% of the surface area of intracortical arterioles [115]. Astrocytes cause vasodilation under physiological conditions via calcium-dependent activation of BK channels and potassium release from astrocytic endfeet [116]. As long as the local increase in extracellular potassium concentration remains below 20 mM, BK channel-mediated potassium release from astrocytic endfeet activates inward rectifier potassium channels on the side of vascular myocytes, which hyperpolarizes their plasma membrane, closes voltage-gated L-type calcium channels. and leads to vasodilation. However, when the intraastrocytic calcium response doubles from the normal 300–400 nM to 700–800 nM at the endfeet, astrocyte-mediated vasoconstriction occurs instead of vasodilation [117]. Since BK channels have a 16-fold increased probability of opening when the intraastrocytic calcium response doubles, BK channel activation is greatly increased under these conditions [118]. As a result, the local extracellular potassium concentration in the confined perivascular space may potentially exceed 20 mM. This would lead to (1) depolarization of vascular myocytes, (2) activation of L-type calcium channels, and thus (3) vasoconstriction [117]. In the context of aSAH and the inverse neurovascular response, this mechanism could be interesting because increased BK channel activity in response to increased activity-induced astrocytic calcium oscillations converted vasodilation to vasoconstriction in neocortical slices from rats that had previously undergone experimental SAH [113, 119].

**Fig. 6:**
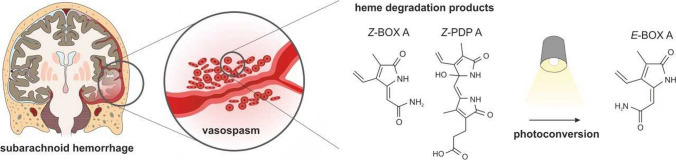
Subarachnoid hemorrhage (SAH) is associated with hemorrhage into the cerebrospinal fluid space. In 85% of cases, SAH results from a rupture of an aneurysm at a basal cerebral artery. Delayed cerebral ischemia is the most important in-hospital complication after aneurysmal SAH (aSAH) and can significantly worsen the prognosis of affected patients [221]. In addition to spreading depolarizations (SD), increased vascular tone and altered neurovascular reactivity, particularly of arteries and arterioles in the cerebral cortex, play an important role in the pathogenesis of DCI. It is assumed that these vascular changes are caused by factors of hemolysis such as higher-order heme degradation products. These include both propentdyopents (PDPs) and bilirubin oxidation end products (BOXes). In addition to their occurrence in the cerebrospinal fluid of aSAH patients, a vasoconstrictive effect on cerebral blood vessels has been demonstrated under in vitro and in vivo conditions in mouse models. The structural-chemical elucidation identified individual isomers within the substance classes of PDPs and BOXes, which can exist in a *Z* and *E* configuration. In the chemical conversion, UV light and visible light are involved. In the specific example of *Z*-BOX A, photoconversion into *E*-BOX A was accompanied by a loss of the vasoconstrictive effect

However, it is not trivial to reconcile this BK channel-based hypothesis of inverse neurovascular responses with the effects of the degradation products of the heme molecule (HDPs). In addition to the tetrapyrroles heme, biliverdin, and bilirubin, HDPs also include the non-enzymatic breakdown of bilirubin formed under the influence of inflammatory processes and elevated concentrations of reactive oxygen species [120, 121]. The resulting regio isomers are divided into two substance classes: the dipyrrole propentdyopents (PDPs) as intermediates of direct cleavage of the bilirubin ring structure [122] and monopyrrole bilirubin oxidation end products (BOXes) as final derivatives of bilirubin and PDP cleavage [123, 124] (Fig. 6). The structural-chemical elucidation identified four regio isomers within the substance class of PDPs (PDP A1/A2, PDP B1/B2) and four further regio isomers within the BOXes cohort (BOX A, BOX B, BOX C, and BOX D). The A and B isomers of PDPs and BOXes identified so far can exist in two different configurations, the *Z* and *E* configuration as a result of a rotation at the exocyclic double bonds [125]. In studies investigating the amount of HDPs in cerebrospinal fluid (CSF), serum/plasma, and bile, exclusively *Z*-configured BOXes and PDPs were detected. This leads to the conclusion that the naturally occurring *Z*-configuration of PDPs and BOXes is the thermodynamically most stable form, while the exposure to UV light and visible light induces the conversion of *E*-isomers, which leads to higher-energetic, i.e., more labile states [126]. Using high-performance liquid chromatography coupled to mass spectrometry *Z*-BOXes and *Z*-PDPs were quantified in the CSF of aSAH patients resulting in nanomolar concentrations, whereas these compounds were barely detectable in the control group [127]. In aSAH patients, the concentration of PDPs exceeded that of BOXes many times over. This suggests that especially PDPs have the potential to influence the pathogenesis of increased cerebrovascular tone after aSAH.

Data from Hou et al. [128] suggested that BK channels may be a target of HDP-mediated action on the neurovascular unit. Using a transgenic mouse model with a conventional knockout of the BK channel-encoding Slo1 gene, the vasoactivity of HDP isomers was investigated as a function of BK channel expression under in vitro and in vivo conditions. Acute vasoconstriction of cerebral arterioles was detected in both in vitro mouse brain slices and in vivo two-photon imaging. The subsequently tested PDPs also had a comparable vasoconstrictive effect on cerebral arterioles. The vasoconstrictor effect was dependent on the expression of the BK channel. In Slo1 knockout mice without a functional BK channel, there was no change in diameter [127, 129]. In addition, the *E*-configured BOX A regio isomer failed to induce a vasoconstrictive effect under in vitro and in vivo conditions, which underlines the dependence on the molecular structure [125].

In addition to these diameter data from acute experiments, Joerk et al. [127] investigated cerebral perfusion in mice using contrast-enhanced high-resonance MRI (9.4 T) over a period of up to 14 days. After intrathecal injection of autologous blood via the cisterna magna (positive control of an experimentally induced SAH) as well as for *Z*-PDPs, a delayed cortical perfusion delay, reversible after 14 days, could be demonstrated as a functional correlate in the mouse model. The effect reached its peak between days 3 and 7 after the intervention. The *Z*-PDP concentrations used were based on concentrations in human CSF samples.

In summary, the model of the inverse (vasoconstrictive) response to functional activation after experimental SAH by Koide et al. [113] is based on the assumption of increased BK channel activity, while the model of chronic vasoconstriction by increased *Z*-PDP by Joerk et al. [127] after experimental SAH is based on reduced BK channel activity. This real or apparent contradiction requires further investigation.

## Neuroinflammation

The neurovascular unit (NVU) is comprised of vascular cells (endothelium, vascular smooth muscle cells, pericytes), glia (astrocytes, microglia, oligodendrocytes), and neurons. It is assumed that the extravasated blood in the subarachnoid space has toxic and inflammatory effects that involve all NVU elements. According to the outside-in principle, the strongest effects are to be expected directly at the interface to the subarachnoid blood. Fitting to the fact that these effects are central to the mechanism of damage, the maximum ischemic brain damage develops in the cortex covered by subarachnoid clots [8].

Blood components pass from the clot via perivascular, glymphatic channels into the parenchyma of the cortex and also into the CSF, where they can be measured together with factors involved in neuroinflammation. For example, multiple studies have shown that inflammatory cytokines are significantly upregulated in CSF of aSAH patients, including TNF-α, soluble TNF receptor 1, IL-6, IL-8, and IL-1 receptor antagonist [130–133]. Several studies have reported a correlation of TNF-α, IL-6, and IL-8 with the development of DCI [134–137]. Moreover, the increase of inflammatory cytokines is also accompanied by an activation of complement components in human CSF after aSAH. Literature suggests that the complement activation in parallel with the formation of membrane attack complex may contribute to angiographic vasospasm, while depletion of complement decreased angiographic vasospasm after experimental SAH [138–141].

Next to the release of cytokines and complement activation in the subarachnoid space, the blood constituents recruit immune cells to the site of aneurysm rupture, with emerging evidence pointing towards involvement of these innate immune cells in the inflammatory processes after SAH [142–146]. Neutrophils are recruited to the endothelium as part of the intravascular inflammation in the acute stage of SAH, mediated by ICAM-1 on endothelial cells and PSGL-1 on neutrophils, which could contribute to delayed injury [147]. In experimental SAH, neutrophils have been implicated in causing early hypoperfusion [148]. Neutrophil count (or its ratio to other cell types) may predict aSAH patients at risk for DCI and poor outcome [149–152].

Complementary studies have revealed further inflammatory signaling within cerebral vessels after SAH. The upregulation of pro-inflammatory mediators such as IL-1, IL-6, and MMP-9 has been linked to the activation of the MEK-ERK1/2 signaling pathway in isolated cerebral vessels from rat models [153]. Surface proteins on leukocytes, including Toll-like receptor-4 (TLR-4), TRIF, and MyD88, have been implicated in the mediation of neuronal apoptosis and increased vascular tone through the NFkB and IRAK4 pathway [154]. Interestingly, inhibition of vascular adhesion protein-1 (VAP-1) led to reduced neutrophil trafficking, and subsequently, an improvement in SAH-associated cerebrovascular dilating dysfunction [155, 156].

A relatively new, emerging concept as part of the inflammatory activation is the release of so-called neutrophil extracellular traps (NETs), which are fibril matrixes containing DNA, granular proteins, and histones, released by neutrophils upon stimulation [157]. NETs have been shown to be involved in various immune reactions [146, 158, 159]. After SAH, they begin to be released into the subarachnoid space ipsilateral to the hemorrhage shortly after its occurrence and gradually accumulate in the parenchyma over time, even at remote compartments [160]. Both depletion of neutrophils by anti-Ly6G as well as DNase I treatment reduced NET formation and microthrombi, and ameliorated neuronal injury after experimental SAH in mice [161]. Similarly, Zeng et al. demonstrated that NET accumulation occurs after SAH and found that inhibition of NET formation by the PAD4 antagonist GSK484 and by DNase I inhibited NET-associated neuroinflammation [162]. More recently, neutrophils and NETs have been observed to cause microvascular occlusion after experimental SAH in mice [163]. It has also been observed that inhibition of neutrophils (through depletion or other targets) leads to a decrease in delayed vasospasm and improved memory function in mice [164–166].

**Fig. 7:**
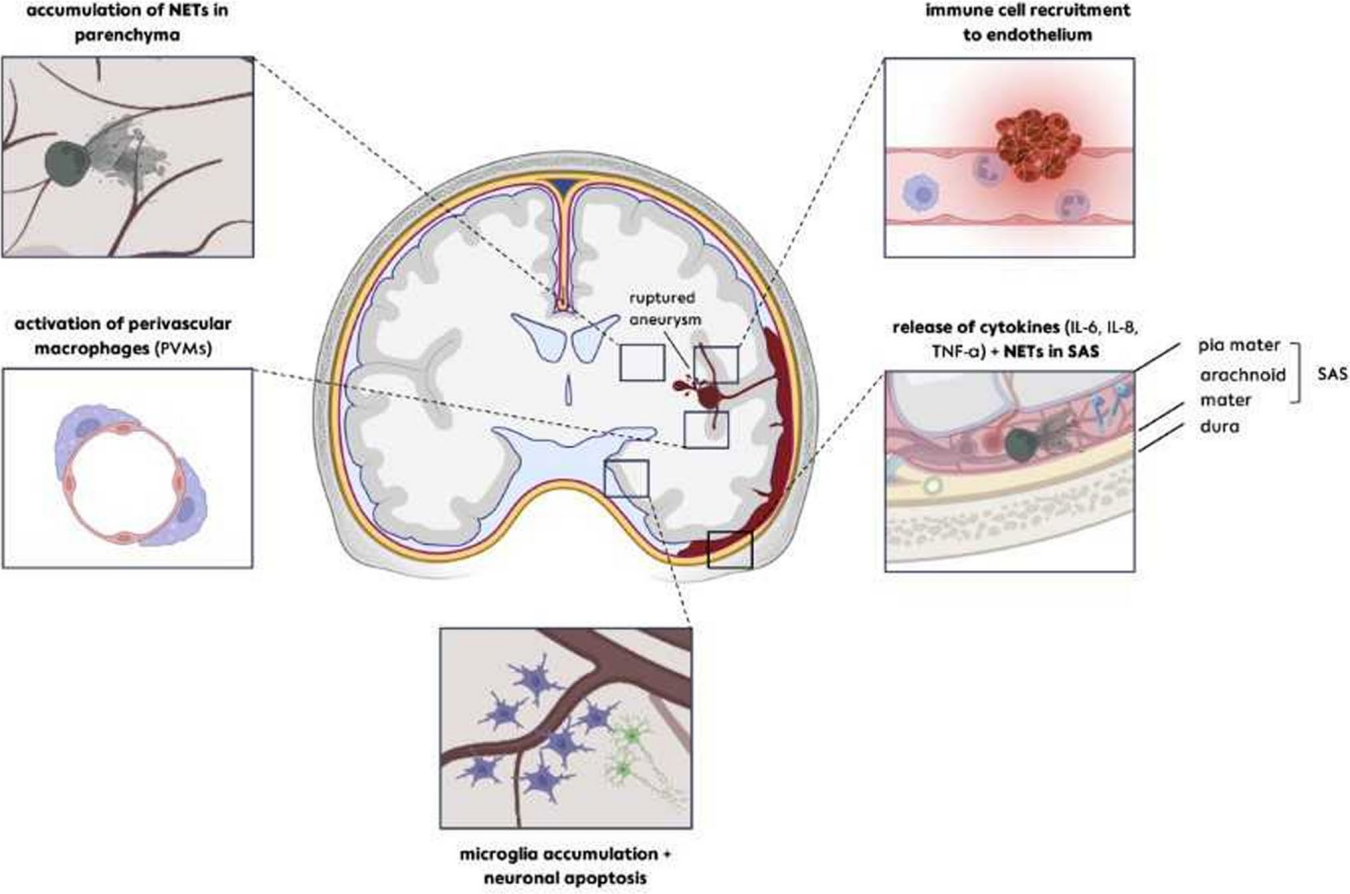
After the rupture of an aneurysm, blood leaks into the subarachnoid space, which is located between the pia mater and the arachnoid membrane and is therefore practically directly adjacent to the cortical brain tissue. In addition, the blood can also reach the parenchyma of the cortex via glymphatic channels. The spatial proximity between blood and cortex tissue could be of great importance, as almost 70% of focal brain damage detected in neuroimaging after aneurysmal subarachnoid hemorrhage (aSAH) involves the cortex. Following aSAH, a sequence of inflammatory reactions unfolds from the outer to the inner regions: in the brain’s microvascular system, there is a noticeable clustering of neutrophil granulocytes on the endothelium, driven by ICAM-1 on endothelial cells and PSGL-1 on neutrophils. Neutrophil extracellular traps (NETs) are released into the subarachnoid space ipsilateral to the hemorrhage shortly after the initial hemorrhage and gradually accumulate in the parenchyma over time, spreading to cortical and periventricular compartments distant from the maximum hemorrhage localization. Microglial accumulation and activation occur approximately 1 week following the injury, marked by their release of pro-inflammatory cytokines such as Il-6, TNF-alpha, and Il-1alpha/beta. This surge in microglial activity coincides with microglial-neuronal interactions in the cortex, leading to neuronal/axonal damage that is most pronounced from day 7 to day 14 after the initial hemorrhage

As part of the outside-in activation of the inflammatory cascade, resident microglia, which are the main immune effector cells in the brain, accumulate near the site of the vascular rupture in experimental rodent studies [145]. Figure 7 depicts the current concept of the cellular inflammatory response after SAH. Accumulation and activation of microglia peak between 9 to 14 days following the initial hemorrhage, marked by their production of pro-inflammatory cytokines such as IL-6, TNF-α, and IL-1alpha/beta [145]. Microglial activation is accompanied by interactions with neurons that can result in neuronal degeneration after experimental SAH. Overall, however, microglia may play a Janus-faced role as a promoter of inflammation on the one hand and as an anti-inflammatory, protective factor, and eliminator of blood degradation products after SAH on the other [167, 168]. SD clusters may induce additional microglial activation after aSAH subsequent to neuronal NLRP3 inflammasome activation [169]. Toll-like receptors TLR2/4, the potential receptors of the damage-associated molecule pattern HMGB1 [170], appear to be involved in this. Although often discussed in the context of migraine with aura, SD-induced neuroinflammation might be even more relevant to conditions such as aSAH, where there are multiple, longer, and more dangerous SDs [2, 20, 99, 171] (Fig. 3). SDs are also involved in the upregulation of proinflammatory cytokines such as IL-1, which has been detected in increased concentrations in the cerebral cortex and CSF in connection with SAH [172]. These increased IL-1 levels catalyze the activation of matrix metalloproteinases (MMPs), which can lead to a dysfunction of the blood–brain barrier (BBB) and promote further neuroinflammatory reactions [173]. Moreover, IL-1 is implicated in contributing to microvascular dysfunction by promoting sarcoplasmic calcium release, myosin light chain phosphorylation, and vasoconstriction [174]. It may be added that SDs even open the BBB independently of SAH by inducing caveolin-1-dependent endothelial transcytosis [175] and activating and upregulating MMP-9 [176].

Next to microglia, perivascular macrophages (PVMs) are also intricately involved in the immune response after SAH. They are strategically located on the walls of blood vessels within the brain, where they perform functions akin to microglia, acting as key players in the immune response [177–180]. In addition to these shared roles, PVMs also undertake unique tasks, including the clearance of waste from the perivascular space [177, 181, 182], BBB maintenance [183], rCBF regulation [184], modulation of endothelial function [185], and activation of sympathetic nerves through the production of prostaglandin E2 and cyclooxygenase 2 [186–191]. These diverse functions underscore the importance of PVMs in maintaining brain homeostasis. However, under pathological conditions, such as after SAH, PVMs can be strongly activated, leading to an acceleration of brain inflammation and potentially contributing to DCI [192]. In SAH, sympathetic nerve activation is associated with worse prognosis and clinical severity [193, 194]. For example, PVMs could influence DCI by mediating systemic inflammation and sympathetic nerve activation. To further elucidate the mechanisms and roles of PVMs, researchers have employed clodronate liposomes to selectively deplete PVMs and observe the resulting differences compared to control groups. Notably, intracerebroventricular administration of clodronate has been shown to selectively deplete PVMs without affecting microglia and circulating macrophages [195]. Several studies have shown that intracerebroventricular administration of clodronate improves the outcome of SAH in animal models [192, 196–199].

As mentioned, astrocytes are another important player. They not only regulate rCBF, are an essential BBB component, and maintain synaptic homeostasis, but also appear to contribute to the inflammatory response [200, 201]. Interestingly, ex vivo and in vitro studies have shown that SDs affect the characteristics and inflammatory response of astrocytes, including an increase in their pro-inflammatory cytokine production such as TNF-α, IL-1β, IL-6, and MMP-9, particularly when exposed to oxyhemoglobin [202, 203]. Concurrently, astrocytes produce neurotrophic factors and upregulate MyD88 and IL-33 after SAH, contributing both to the inflammatory response regulation and to neuroprotection, thereby helping to maintain the integrity of the BBB [204, 205].

## Conclusion

Overall, SDs are increasingly recognized not only as a clinical marker for DCI [2], but also as the crucial mechanism underlying the development of the characteristic cortical infarcts after aSAH. Whether they are harmless, or cause infarcts, most likely depends on whether the complex neuroglial, neurovascular, and neuroimmunological regulatory circuits that normally protect the cortex and allow rapid repolarization after SD are locally disrupted by red blood cell products. Possible treatment targets are not only an improved elimination of the triggering agent (i.e., the extravascular blood [206–211]), and other conditions that may contribute to SD, but also a correction of the dysfunctional regulatory circuits that are downstream of SD and render it truly dangerous. If these regulatory circuits are no longer able to rescue the neurons from the SD state, they will inevitably perish. In this way, the cortically localized mechanisms of infarct development in the area of the subarachnoid blood clots discussed here and the chronic increase in tone in the various segments of the arterial tree are also not mutually exclusive, but complementary pathomechanisms after aSAH. Overall, we therefore argue that therapeutic combination approaches should also be pursued further [3].

## Data Availability

No datasets were generated or analysed during the current study.

## Abbreviations

AC: Alternating current
aSAH: Aneurysmal subarachnoid hemorrhage
BBB: Blood-brain barrier
BK channel: Large conductance calcium-activated potassium channel (big potassium)
BOXes: Monopyrrole bilirubin oxidation end products
CSF: Cerebrospinal fluid
CT: Computed tomography
DC: Direct current
DCI: Delayed cerebral ischemia
DISCHARGE-1: Depolarizations in ischemia after subarachnoid hemorrhage-1
DSA: Digital subtraction angiography
EBI: Early brain injury
ECI: Early cerebral ischemia
ECoG: Electrocorticography
HDP: Degradation products of the heme molecule
HMGB1: High-mobility-group-protein B1
ICH: Intracerebral hemorrhage
ICP: Intracranial pressure
IQR: Interquartile range
MCA: Middle cerebral artery
MCAO: Middle cerebral artery occlusion
MRI: Magnetic resonance imaging
MWRST: Mann-Whitney rank sum test
Na,K-ATPase: Sodium pump
NET: Neutrophil extracellular trap
NMDAR: N-methyl-d-aspartate receptor
NO: Nitric oxide
NOS: Nitric oxide synthase
NUP: Negative ultraslow potential
NVU: Neurovascular unit
PDP: Dipyrrole propentdyopents
PTDDD: Peak total spreading depolarization-induced depression duration of a recording day
PTDDD_delayed_: Peak total spreading depolarization-induced depression duration of a recording day in the delayed neuromonitoring period
PVM: Perivascular macrophage
rCBF: Regional cerebral blood flow
SAH: Subarachnoid hemorrhage
SD: Spreading depolarization
TCD: Transcranial Doppler sonography
TDDD: Total spreading depolarization-induced depression duration of a recording day
VAP-1: Vascular adhesion protein-1
